# Complete Genome Sequences of Two Multidrug-Resistant *Mycobacterium tuberculosis* Strains Isolated in Guiyang, China

**DOI:** 10.1128/genomeA.01257-17

**Published:** 2018-01-04

**Authors:** Zhenghong Chen, Weizheng Ou, Xiangyu Fan, Guzhen Cui, Qiong Wang, Qiang Li, Rong Sun, Xiaojuan Wu, Wan Qin, Yan Wang

**Affiliations:** a Microbiology Department, Key Laboratory of Medical Microbiology and Parasitology, Guizhou Medical University, Guiyang, China; b Clinical Laboratory, Guiyang Public Health Clinical Center, Guiyang, China; c School of Biological Science and Technology, University of Jinan, Jinan, China; d Shanghai ZJ Bio-Tech, Ltd., Shanghai, China

## Abstract

We identified the genome sequences of two *Mycobacterium tuberculosis* isolates. They were resistant to rifampin and isoniazid, as determined by the agar proportion method, but were susceptible to isoniazid, as determined by the DNA array method. The genome sequences showed that a* katG* deletion led to the false diagnosis of isoniazid resistance by DNA array.

## GENOME ANNOUNCEMENT

Tuberculosis (TB) remains one of the leading causes of morbidity and mortality worldwide. Drug-resistant TB, especially multidrug-resistant TB (MDR-TB), is one of the main obstacles to controlling TB. MDR-TB is caused by *Mycobacterium tuberculosis* that is resistant to both rifampin (RFP) and isoniazid (INH). Appropriate MDR-TB management requires drug susceptibility testing to guide treatment regimens. The usual turnaround time for phenotypic *M. tuberculosis* drug susceptibility testing is about 8 to 10 weeks, and performing this testing often leads to delays in the initiation of appropriate therapy. Therefore, genotypic methods for rapid detection of drug-resistant *M. tuberculosis* strains are required.

DNA arrays targeting the *rpoB* gene (loci 511, 513, 516, 526, and 533), *katG* gene (locus 315), and *inhA* gene (locus 15) are now widely employed to detect RFP or INH resistance. From 2014, in the Guiyang Public Health Clinical Center, screening of these mutations was performed with DNA arrays obtained by using the *M. tuberculosis* drug resistance detection array kit (CapitalBio Corporation, China). However, the concordance between phenotypic and genotypic testing was not 100%.

Here, the complete genome sequences of two *M. tuberculosis* strains (D8788 and D7070) are described. These strains showed resistance to the four first-line anti-TB drugs (RFP, INH, streptomycin, and ethambutol), which was determined by the agar proportion method, but were INH susceptible according to detection by DNA array.

Genomic DNA was isolated using the Wizard genomic DNA purification kit (Promega, Madison, WI, USA). A DNA library was prepared using a 250-bp paired-end Nextera XT DNA library prep kit (Illumina, San Diego, CA, USA), quantified by using a Labchip GX Touch II (PerkinElmer, MA, USA), and sequenced on a Miseq platform (Illumina). Raw data obtained from next-generation sequencing were mapped to the reference genome of H37Rv (NCBI reference sequence NC_000962), and assembled using Velvet 0.7.55-1 (1). Gap closing was carried out by Sanger sequencing. Comparative genomics was employed for these two isolates and the other genome sequences of *M. tuberculosis* on NCBI, for example H37Rv (a reference strain, which is susceptible to RFP and INH) (2) and CCDC5180 (which is resistant to all four first-line drugs) (3).

Finally, the complete genome sequence of D8788 was noted to be 4,304,863 bp, with 65.51% GC content, and that of D7070 was noted to be 4,280,826 bp, with 65.54% GC content. The genome length of these two strains was about 100 kb shorter than that of H37Rv (2) and CCDC5180 (3). Interestingly, the BLAST search of *katG* sequence (2,222 bp) of H37Rv and CCDC5180 on the genome of strain D7070 showed that no hits were found. Additionally, the score of strain D8788 was only 1,586 bits (965 bp), suggesting that the *katG* deletion occurred in these two strains, leading to the false diagnosis of INH resistance as determined by DNA array.

*M. tuberculosis* is largely clonal (4, 5). Studies on MDR *M. tuberculosis* have revealed mutations in hot-spot regions, whereas additional studies are required to identify mutations occurring beyond the hypervariable regions or other molecular mechanisms underlying resistance. Although the DNA array has significant value and allows rapid diagnosis of INH-resistant TB, it is apparent that further improvements are required.

### Accession number(s).

This whole-genome shotgun project has been deposited at DDBJ/ENA/GenBank under the accession numbers NXGF00000000 and NXGG00000000. The versions described in this paper are the first versions.
