# Correction for Chen et al., “Complete Genome Sequences of Two Multidrug-Resistant Mycobacterium tuberculosis Strains Isolated in Guiyang, China”

**DOI:** 10.1128/MRA.00081-20

**Published:** 2020-03-05

**Authors:** Zhenghong Chen, Weizheng Ou, Xiangyu Fan, Guzhen Cui, Qiong Wang, Qiang Li, Rong Sun, Xiaojuan Wu, Wan Qin, Yan Wang

**Affiliations:** a Microbiology Department, Key Laboratory of Medical Microbiology and Parasitology, Guizhou Medical University, Guiyang, China; b Clinical Laboratory, Guiyang Public Health Clinical Center, Guiyang, China; c School of Biological Science and Technology, University of Jinan, Jinan, China; d Shanghai ZJ Bio-Tech, Ltd., Shanghai, China

## AUTHOR CORRECTION

Volume 6, no. 1, e01257-17, 2018, https://doi.org/10.1128/genomeA.01257-17. Page 1, line 30: “D8788” should read “D7070.”

Page 1, line 31: “D7070” should read “D8788.”

Page 2, line 3: “D7070” should read “D8788.”

Page 2, line 4: “D8788” should read “D7070.”

